# Evidence-based supportive care for patients undergoing radiotherapy: Recommendations from the updated German national S3 guideline for oncological patients

**DOI:** 10.1007/s00066-026-02532-1

**Published:** 2026-04-16

**Authors:** Cas Stefaan Dejonckheere, Jürgen Heide, Jiadai Zou, Frank Zimmermann, Leonard Christopher Schmeel, Ulrike Höller, Lukas Käsmann

**Affiliations:** 1 Department of Radiation Oncology, University Hospital Bonn, Bonn, Germany; 2 Radiation Oncology Harburg, Hamburg, Germany; 3 Department of Radiation Oncology, University Hospital Leipzig, Leipzig, Germany; 4 Department of Radiation Oncology, University Hospital Basel, Basel, Switzerland; 5 Department of Radiation Oncology, Cologne Municipal Hospitals, University Hospital of the University of Witten-Herdecke, Cologne, Germany; 6 Deutsche Gesellschaft für Radioonkologie (DEGRO), Berlin, Germany; 7 Department of Radiation Oncology, LA-Regio Kliniken Klinik Landshut-Mitte, Landshut, Germany; 8 Department of Radiation Oncology, University Hospital Munich (LMU), Munich, Germany

## Introduction

The prevention and management of radiation-induced toxicities represents an integral part of modern radiation oncology. The German S3 guideline “Supportive Therapy in Oncological Patients” was originally published in 2016 by the Association of the Scientific Medical Societies in Germany (*Arbeitsgemeinschaft der Wissenschaftlichen Medizinischen Fachgesellschaften*, AWMF). Based on the latest evidence, its recommendations were revised most recently in April 2025 [1]. Existing chapters were reviewed, including those on tumor treatment-induced anemia, antiemesis, the use of granulopoietic growth factors, skin and mucosal toxicities, treatment-induced diarrhea, peripheral neurotoxicity, osseous complications, extravasation management, and supportive treatment in radiation oncology. In addition, the guideline has been expanded to cover emerging aspects such as autoimmune diseases as treatment-related adverse effects, cardiotoxicity, central neurotoxicity, and radiation-induced toxicities affecting the urogenital tract. In the following, we provide a concise overview of the current guideline update and discuss its evidence-based recommendations for clinical practice.

The guideline also includes a list of changes and new additions (pages 3–10). Also noteworthy are the newly assembled lists of substances that are of limited use or are insufficiently tested (e.g., radiation-induced dermatitis, mucositis, proctitis). These measures should be avoided to spare patients from potential side effects and/or unnecessary effort and costs.

## Skin

The management of acute radiation dermatitis remains controversial, inconsistent, and partially not supported by evidence-based medicine [2]. As general prophylactic measures, washing of skin and hair is explicitly recommended, using mild, skin pH-adjusted products. Skin care with basic creams (e.g., containing 2–5% urea without allergenic substances) is considered good clinical practice and should be performed. Use of deodorant or creams immediately before radiation is now permitted, reflecting a shift from older restrictive practices. Silver sulfadiazine cream is no longer recommended due to insufficient evidence of efficacy and the risk of severe side effects such as toxic epidermal necrolysis. Topical corticosteroids (e.g., mometasone furoate) are recommended in patients with risk factors such as skin dose ≥ 50 Gy, skin folds, large breast volume/obesity, or active smoking. Evidence is strongest for moderate-to-high-potency steroids, and the often-feared adverse effects of their prolonged use (e.g., skin atrophy, striae) are generally not observed. Photobiomodulation therapy, the local application of light from the red or near-infrared spectral range, is now discussed as a prophylactic option. While promising, its use remains institution dependent and costly. Also newly added are barrier film dressings (e.g., Hydrofilm® [Paul Hartmann, Heidenheim, Germany], Mepitel® [Mölnlycke Health Care, Gothenburg, Sweden]) in the context of breast irradiation [3]. Their use for radiation dermatitis prevention in the head and neck region as well as spray-on skin protectants require additional investigation and are currently not recommended.

The treatment of acute radiation dermatitis is primarily symptom oriented, with cooling and topical corticosteroids for inflammatory symptoms. Of note, a list of topical and oral substances that are (strongly) discouraged is provided and should be consulted by caregivers. Treatment of moist desquamation as well as chronic radiation ulcers follows the general rules of wound care [1].

## Central nervous system

Corticosteroids (e.g., dexamethasone) should not be prescribed prophylactically in the context of brain irradiation but can be used for prevention of radiation-related symptoms in intraspinal tumors [4]. Neuroprotectants (e.g., memantine) should not be routinely used for the prophylaxis of radiation-induced neurocognitive late effects. The role of hippocampal avoidance for whole-brain radiotherapy (WBRT), however, is emphasized.

Symptomatic edema during or after radiotherapy should be treated with corticosteroids, using the lowest possible effective dose, continuously adapting to the patient’s symptoms and tapering as soon as possible. However, caution is advised in patients receiving immunotherapy due to negative effects on outcome [5]. In cases of symptomatic brain radionecrosis, treatment with bevacizumab may be considered as an off-label option [6].

## Oral cavity

Radiation-induced oral mucositis remains one of the most frequent side effects of head and neck radiotherapy. Standardized oral care (i.e., gentle cleaning with a soft toothbrush, regular rinsing, avoiding irritants, fluoridation, and close monitoring during and after treatment) is reaffirmed as the cornerstone of prevention. Among pharmacologic strategies, benzydamine is now explicitly recommended during radiotherapy, with conditional use in radiochemotherapy. Oral glutamine may be considered, as supported by multiple randomized trials [7]. Honey is recognized as a non-pharmacologic prophylactic option, whereas the costlier Manuka honey, prophylactic antimicrobial rinses, sucralfate, misoprostol, curcumin, and gabapentin are discouraged due to lack of evidence [8]. Intraoral photobiomodulation therapy, similar to radiation dermatitis, has been acknowledged as a preventive modality that may be used.

For treatment of mucositis, the emphasis lies on structured pain management including topical and systemic analgesics, with opioid mouth rinses as an additional option. Management of treatment-related xerostomia includes saliva substitutes and sialogogues like pilocarpine.

## Gastrointestinal

Acute radiation-induced side effects in the gastrointestinal tract typically present as mucosal inflammation with diarrhea, abdominal cramps, tenesmus, and occasionally rectal bleeding. Pathophysiologically, radiation-induced epithelial damage leads to impaired absorption, intestinal barrier dysfunction, and subsequent inflammation. Acute proctitis is most commonly characterized by rectal pain, diarrhea, and the presence of mucus or blood in the stool.

According to the updated guideline, topical aminosalicylates (e.g., mesalazine, olsalazine), rectal misoprostol, sucralfate (oral or enema), hydrocortisone foam, butyrate, and rectal epinephrine should *not* be used for the prophylaxis of acute radiation-induced proctitis. Likewise, rectal application of aloe vera cannot be recommended or rejected due to insufficient evidence [9]. The implementation of image-guided and intensity-modulated radiotherapy has been shown to significantly reduce the risk of both acute and chronic proctitis; bellyboards can be avoided [10]. Based on the results of one larger randomized trial (*n* = 166), a high-fiber diet should be considered for prophylaxis of radiotherapy-induced diarrhea [11]. The impact of other specific dietary interventions on the prevention of diarrhea resulting from enteritis or proctitis has only been assessed in small, heterogeneous randomized studies and cannot be recommended for routine use, in contrast to current practice. The role of the intestinal microbiome in supportive care is currently under investigation. Several randomized trials demonstrated that oral administration of *Lactobacillus* (in combination with *Bifidobacterium*) may reduce proctitis symptoms during pelvic radiotherapy [12, 13]. For prevention of radiation-induced diarrhea, probiotics containing *Lactobacillus acidophilus* or *Bifidobacterium bifidum* may be beneficial. Nevertheless, caution is required in immunocompromised patients, as the use of probiotics may lead to a potentially increased risk of sepsis. These concerns, however, are based solely on blood culture findings from case reports, with no evidence of associated fatalities based on the randomized evidence.

## Genitourinary

This is a newly added section in the 2025 guideline revision, addressing a previously underrepresented topic in supportive radiation oncology. Exclusion of other causes (e.g., urinary tract infection, stones) is emphasized before attributing symptoms to radiation. Pre- or rehabilitation with supervised pelvic floor training can reduce lower urinary tract symptoms. Obstructive symptoms can be prevented or treated with α‑adrenoceptor antagonists (e.g., tamsulosin). Anticholinergic agents (e.g., trospium chloride) can be useful for overactive bladder symptoms. Evidence, however, mainly stems from the context of brachytherapy for prostate cancer. Nonsteroidal anti-inflammatory drugs (e.g., ibuprofen) and selective cyclooxygenase-2 inhibitors (e.g., celecoxib) can also be considered. Respective side effects and contraindications should be considered. Cranberry (juice or tablets), although frequently associated with urological health, is explicitly not recommended due to lack of efficacy across several trials. Rare and serious urinary side effects (e.g., fistula, stricture, refractory hematuria) should be explored and managed interdisciplinarily.

Radiotherapy for pelvic malignancies, particularly prostate cancer, often leads to erectile dysfunction (ED), which can significantly impair quality of life. Vascular and nerve damage are considered the main causes. The prophylactic use of PDE5 inhibitors (e.g., sildenafil) provides no adequate benefit and should thus not be prescribed to prevent ED. As first-line therapy for ED after radiotherapy, PDE5 inhibitors are, however, recommended, as they improve erectile function in about two thirds of affected patients.

Pelvic radiotherapy in female patients can cause acute and long-term vaginal side effects such as stenosis, atrophy, and fibrosis, with subsequent dyspareunia. In addition, radiation exposure of the ovaries may have an impact on hormone levels. Vaginal dilators and lubricants can be used both prophylactically and therapeutically, whereas topical estrogens are suitable for symptom management. The latter, however, remains controversial in patients with endometrial cancer due to uncertain effects on tumor recurrence [14]. Careful counseling on vaginal care and strategies to reduce anxiety are essential during follow-up to enable pain-free intercourse and prevent adhesions.

## Hyperbaric oxygen therapy

Hyperbaric oxygen therapy (HBOT) encompasses the breathing of 100% O_2_ in an enclosed space at an increased pressure, enabling optimized hemoglobin saturation and O_2_ diffusion in tissues. This is of interest in the context of certain late radiation-induced effects, which are usually characterized by hypoxia and fibrosis. In recent years, the number of high-quality trials has increased, whereby HBOT may be used for chronic proctitis, chronic cystitis, and as an adjunct to resective surgery for complicated or extensive surgery for mandibular osteoradionecrosis [15, 16]. The burden of treatment (usually 30–40 sessions of 90–120 min each) should be discussed with patients.

## Management of chemotherapy-related toxicities

Anemia is frequently observed in oncological patients, particularly during concurrent radiochemotherapy. Beyond its high prevalence, anemia is a negative prognostic marker in several tumor entities, as reduced hemoglobin levels impair oxygen delivery and may thus compromise the effectiveness of radiotherapy [17]. The updated guideline recommends considering red blood cell (RBC) transfusion in patients with chemotherapy-induced anemia when hemoglobin levels fall below 10 g/dL (6.2 mmol/L). For hospitalized patients with chronic anemia whose clinical status and laboratory parameters are closely monitored, initial transfusion should generally consist of a single RBC unit. To reduce the frequency of transfusions, the administration of iron—particularly intravenous iron—may be employed after measuring iron status (e.g., serum iron, ferritin). While this strategy has been shown to improve the hematological response, evidence for a consistent benefit in terms of quality of life remains limited [18].

Granulocyte colony-stimulating factor (G-CSF) should not be administered solely in response to afebrile neutropenia following cancer therapy. Its prophylactic use is indicated only when the risk of febrile neutropenia is ≥ 20%, or 10–20% in the presence of additional individual risk factors such as advanced age (i.e., > 65 years), poor performance status, significant comorbidities (e.g., advanced chronic obstructive pulmonary disease [COPD] or heart failure), symptomatic tumor burden, or prior systemic therapy as well as in the presence of laboratory abnormalities such as anemia, lymphocytopenia (< 700/µL), hypoalbuminemia, or hyperbilirubinemia.

The chapter addressing chemotherapy-induced nausea and vomiting (CINV) has undergone substantial revisions. For patients receiving highly emetogenic treatments such as single-day cisplatin-based chemotherapy, prophylaxis should include a 5-HT_3_ receptor antagonist, an NK_1_ receptor antagonist, and dexamethasone. In addition, the off-label use of olanzapine (5 mg) has been shown to further improve antiemetic efficacy and can therefore be considered.

A new mandatory recommendation was made for the introduction of a baseline audiological assessment to evaluate potential ototoxicity before initiating cisplatin-based therapy. Furthermore, the guideline now incorporates a new recommendation for the prevention of chemotherapy-induced polyneuropathy (CIPN): prophylactic cryotherapy or compression therapy may be considered in patients receiving taxane-based therapy [19, 20]. For other antineoplastic agents, however, the current evidence remains insufficient. In patients with chemotherapy-induced neuropathic pain, pregabalin should be offered. Symptoms such as numbness or tingling, although frequently reported by patients, are usually not responsive to this pharmacological intervention.

## Conclusion

The 2025 update provides clearer, evidence-based guidance that aligns with current clinical realities. For radiation oncologists, the emphasis lies on evidence-supported prophylactic and, to a lesser extent, therapeutic regimens. The overall approach is patient-centered and aims to improve quality of life by standardizing care across institutions.

## Data Availability

Data sharing is not applicable to this article as no new data were created or analyzed in this study.
